# MOF-derived ternary ZnCo–Ni LDHs for high-energy-density supercapacitors: synergistic effects and enhanced ion transfer

**DOI:** 10.1039/d5ra09868h

**Published:** 2026-04-22

**Authors:** Gaofu Liu, Kunyu Hao, Zhuanyu Liu, Yiwen Tang, Yonggang Wu

**Affiliations:** a School of Physics and Electronic Science, Guizhou Education University Guiyang 550018 P. R. China ygwu0946@163.com; b Institute of Nano-Science and Technology, College of Physical Science and Technology, Central China Normal University Wuhan 430079 China ywtang@ccnu.edu.cn +86-27-67861185 +86-27-67867947

## Abstract

The development of electrode materials that simultaneously possess high energy density and excellent cycling stability remains a critical challenge hindering the widespread application of supercapacitors. Consequently, traditional binary layered double hydroxides (LDHs) suffer from nanosheet stacking and limited active sites. To address this issue, this paper proposes an innovative strategy to construct ternary LDHs using metal–organic frameworks (MOFs) as precursor templates. Three-dimensional nanoflower-like ZnCo–Ni LDHs (ZCN-LDHs) were fabricated on a ZnCo-MOF template *via in situ* nickel ion introduction and an optimized solvothermal process. This unique 3D hierarchical architecture effectively inhibits the stacking of nanosheets and provides abundant ion transport pathways. Benefiting from the proposed synergistic interplay among Zn, Co, and Ni ions within the unique 3D architecture, the ZCN-LDHs-180_2h_ electrode delivers a high specific capacity of 2288 F g^−1^ at 1 A g^−1^. This enhanced performance is hypothesized to originate from the combined effects of improved electronic structure and increased redox activity, as suggested by electrochemical impedance spectroscopy (EIS) and cyclic voltammetry (CV) analyses. Furthermore, an asymmetric supercapacitor (ASC) assembled with ZCN-LDHs-180_2h_ and commercial activated carbon (AC) achieves an energy density of 39.1 Wh kg^−1^ at a power density of 750 W kg^−1^.

## Abstract 2

The development of electrode materials that simultaneously possess high energy density and excellent cycling stability remains a critical challenge hindering the widespread application of supercapacitors.

## Graphical abstract

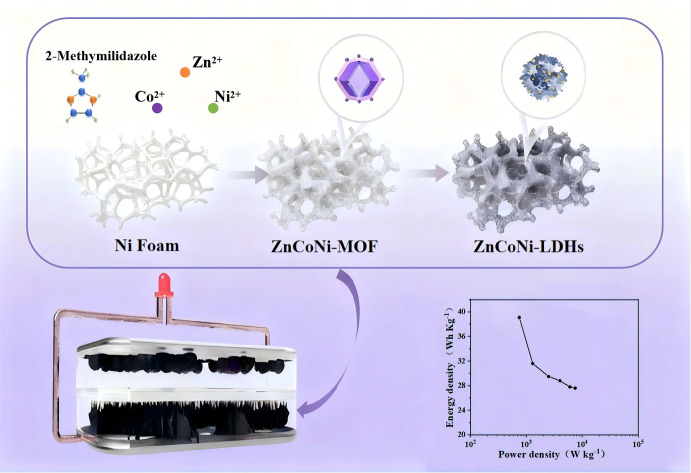

## 1. Introduction

With the continuous increase in global energy demand and growing environmental concerns, the development of highly efficient and stable electrochemical energy storage devices has become a research priority in the energy field.^1–5^ Supercapacitors have attracted considerable attention in fields such as portable electronics and new energy vehicles, owing to their high power density, rapid charge–discharge capability, and exceptional cycling stability.^6,7^ In particular, electrode materials, as the core energy storage medium, directly determine the energy and power density of the devices. Therefore, the design and optimization of electrode material structures are crucial for enhancing the overall performance of supercapacitors.^8–12^

Layered double hydroxides (LDHs), with their unique structure, adjustable chemical composition, and abundant redox active sites, have become an ideal choice for supercapacitor electrode materials. However, traditional binary LDHs generally suffer from limited specific surface area, long ion diffusion paths, and insufficient cycling stability, making it difficult for them to meet the requirements of high-energy-density storage devices.^13–16^ In recent years, researchers have begun to introduce metal–organic frameworks (MOFs) as precursor templates, leveraging their high specific surface area, porous architecture, and controllable topology to open new avenues for the structural design and performance optimization of LDHs. The resulting MOF-derived materials have shown significant promise in electrochemical energy storage applications.^17–22^ Current research has primarily focused on binary LDHs systems such as NiCo-LDHs and ZnCo-LDHs.^23,24^ Meanwhile, ternary or multi-metal doping strategies have further expanded the performance optimization landscape of LDHs. For instance, studies on NiCoAl-LDHs have demonstrated that Al^3+^ doping effectively enlarges the (003) interlayer spacing.^25^ Liao *et al.*^26^ reported an Fe-doped NiCoFe-LDH with an interlayer spacing of 0.89 nm and enhanced OH^−^ diffusion capability. Additionally, Cu-doped NiCo-LDHs has been shown to reduce the redox potential gap by 0.15 V, thereby effectively suppressing electrode polarization. Besides redox tuning, high-valence cation doping can also modulate the electronic structure. For example, Zr-doped NiCoZr-LDHs optimize electronic structure, as Zr^4+^ shifts Co^2+^ 3d orbitals and lowers charge transfer resistance.^27^ Extending this multi-metal synergy, Zhao *et al.*^28^ developed NiCoMnFe-LDHs, *via* gradient metal doping strategy, achieving 85% capacitance retention at 3.5 V.

This study aims to address the issue of insufficient active site exposure caused by the stacking of conventional LDH nanosheets. This is achieved by leveraging the topological guidance effect of the MOF template and the synergistic effect of multimetal components. Specifically, it is hypothesized that the synergistic effect among Zn, Co, and Ni metal ions contributes to the enhanced electrochemical activity and stability of the material, as suggested by the comparison with binary LDHs.^29^ By optimizing the preparation process, we expect to obtain ternary LDH electrode materials with a three-dimensional nanostructure, leading to significantly improved specific capacitance and cycle performance. Subsequently, in an asymmetric supercapacitor hybrid device assembled with activated carbon (AC) as the negative electrode and ZCN-LDHs-180_2h_ as the positive electrode, a high energy density of 39.1 Wh kg^−1^ is achieved at a power density of 750 W kg^−1^.

## 2. Experimental Section

### 2.1 Nickel mesh treatment

Nickel mesh was cut into pieces, sequentially sonicated in anhydrous ethanol and hydrochloric acid, followed by rinsing and drying.

### 2.2 Materials synthesis

#### 2.2.1 Synthesis of Zn-MOF

75 mmol of 2-methylimidazole and 20 mmol of zinc nitrate hexahydrate (Zn(NO_3_)_2_·6H_2_O) were each dissolved in 75 mL of methanol (≥99.8%) and stirred for 10 min. The two solutions were then mixed and stirred at room temperature for 24 h. The product was centrifuged, washed repeatedly with methanol and collected. Final purification involved vacuum drying at 70 °C for 12 h.

#### 2.2.2 Synthesis of Co-MOF

Synthesized following the same procedure as Zn-MOF, except Zn(NO_3_)_2_·6H_2_O was replaced with 20 mmol of cobalt nitrate hexahydrate (Co(NO_3_)_2_·6H_2_O).

#### 2.2.3 Synthesis of ZnCo-MOF

400 mg of Zn-MOF, 75 mmol of 2-methylimidazole, and 20 mmol of Co(NO_3_)_2_·6H_2_O were separately dissolved in 75 mL of methanol. The resulting solutions were then mixed and stirred at room temperature for 24 h. The precipitate was collected by centrifugation, washed, and finally dried at 70 °C for 12 h.

#### 2.2.4 Synthesis of ZCN-LDHs

100 mg of ZnCo-MOF and 0.1 mol of nickel sulfate hexahydrate (NiSO_4_·6H_2_O) were homogeneously dispersed in 30 mL of methanol and stirred for 30 min. The resulting mixture was then transferred to a 100 mL Teflon-lined stainless-steel autoclave and heated in a convection oven at 180 °C for 2 h. After the autoclave had cooled to room temperature naturally, the solid product was collected by centrifugation, thoroughly washed three times with deionized water and then three times with anhydrous ethanol, and finally dried at 60 °C for 12 h.

#### 2.2.5 Synthesis of ZCN-LDHs series

##### 2.2.5.1 Effect of temperature

To investigate the influence of reaction temperature, a series of syntheses were conducted at 140, 160, 170, 180, and 190 °C for a fixed duration of 2 h. The resulting products were labeled as ZCN-LDHs-140, ZCN-LDHs-160, ZCN-LDHs-170, ZCN-LDHs-180, and ZCN-LDHs-190, respectively.

##### 2.2.5.2 Effect of time

To examine the effect of reaction time, samples were prepared at 180 °C for 0.5, 1, 2, and 3 h. The corresponding products were denoted as ZCN-LDHs-180_0.5h_, ZCN-LDHs-180_1h_, ZCN-LDHs-180_2h_, and ZCN-LDHs-180_3h_, respectively.

##### 2.2.5.3 Effect of Zn/Co/Ni ratio

To optimize the metal ratios, a series of ZnCo–Ni LDHs were synthesized under otherwise identical conditions. The optimal sample, denoted as ZCN-LDHs-180_2h_, was obtained with a Zn : Co : Ni molar ratio of 0.2 : 0.2 : 1. For comparison, additional samples with different metal ratios were also prepared, specifically: 0.2 : 0.2 : 0.5, 0.2 : 0.2 : 1.5, 0.2 : 0.3 : 0.5, 0.2 : 0.3 : 1, 0.2 : 0.3 : 1.5, 0.2 : 0.1 : 0.5, 0.2 : 0.1 : 1, 0.2 : 0.1 : 1.5, 0.1 : 0.2 : 0.5, 0.1 : 0.2 : 1 and 0.1 : 0.2 : 1.5. All syntheses were carried out at 180 °C for 2 hours, and the resulting products were named ZCN-LDHs_1_-180_2h_ through ZCN-LDHs_11_-180_2h_, corresponding to the order listed above, with ZCN-LDHs-180_2h_ (Zn : Co : Ni = 0.2 : 0.2 : 1) representing the optimal ratio.

#### 2.2.6 Preparation of binary LDHs

All samples were prepared *via* a solvothermal method at 180 °C for 2 h with the following reactant combinations:

ZnCo-LDHs (ZC-LDHs): 100 mg of Zn-MOF + 1 mmol of CoSO_4_·6H_2_O.

ZnNi-LDHs (ZN-LDHs): 100 mg of Zn-MOF + 1 mmol of NiSO_4_·6H_2_O.

CoNi-LDHs (CN-LDHs): 100 mg of Co-MOF + 1 mmol of NiSO_4_·6H_2_O.

### 2.3 Electrode preparation and supercapacitor assembly

#### 2.3.1 Cathode preparation

All as-synthesized samples were fabricated into electrodes following the same procedure. Typically, 14 mg of the sample. 4 mg of carbon black, and 2 mg of PTFE were mixed in a 7 : 2 : 1 mass ratio. The mixture was transferred to a ball-milling tube with isopropyl alcohol and thoroughly milled at high speed until a homogeneous slurry was formed. The resulting slurry was then drop-cast onto a pretreated nickel mesh (area ≈1 cm^2^), vacuum-dried at 60 °C for 6 h, and finally pressed to ensure good adhesion and prevent material detachment during electrochemical testing. The optimal sample is denoted as ZCN-LDHs-180_2h_ (Zn : Co : Ni = 0.2 : 0.2 : 1).

#### 2.3.2 Anode preparation

Activated carbon (AC) replaced ZCN-LDHs-180_2h_, with other steps identical to cathode preparation.

#### 2.3.3 Device assembly

The ZCN-LDHs-180_2h_//AC asymmetric supercapacitor (ASC) was fabricated by vacuum-encapsulating ZCN-LDHs-180_2h_ (cathode), AC (anode), 2 M KOH (electrolyte), and NKK-MPF30AC-100 polypropylene membrane (separator).

## 3. Results and discussion

### 3.1 Synthesis and structural characterization

**Scheme 1:**
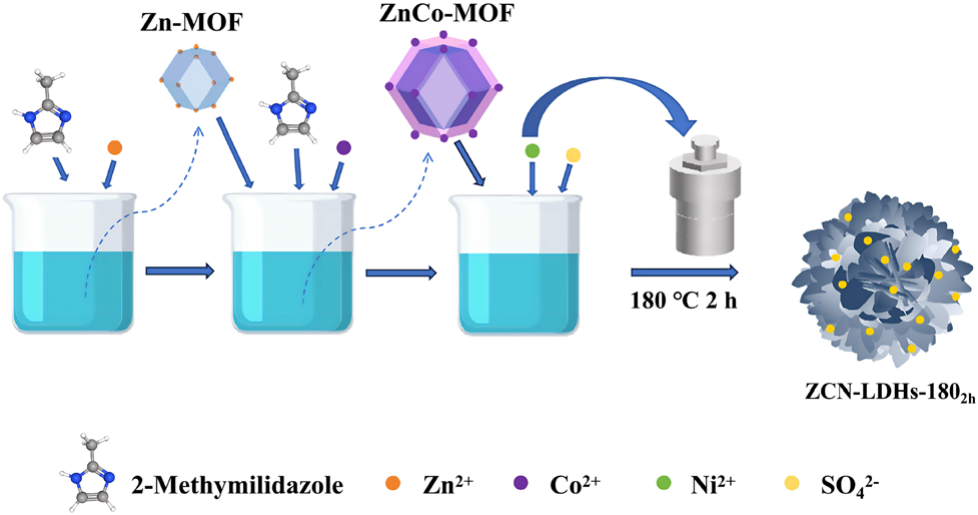
Illustration of the preparation process of ZCN-LDHs-180_2h_.

Scheme 1 illustrates the preparation process. ZCN-LDHs-180_2h_ with a hierarchical structure and nanosheet-petal morphology was prepared *via* a solvothermal method at 180 °C for 2 h, utilizing ZnCo-MOF as a precursor and incorporating Ni ions *in situ*.

**Fig. 1:**
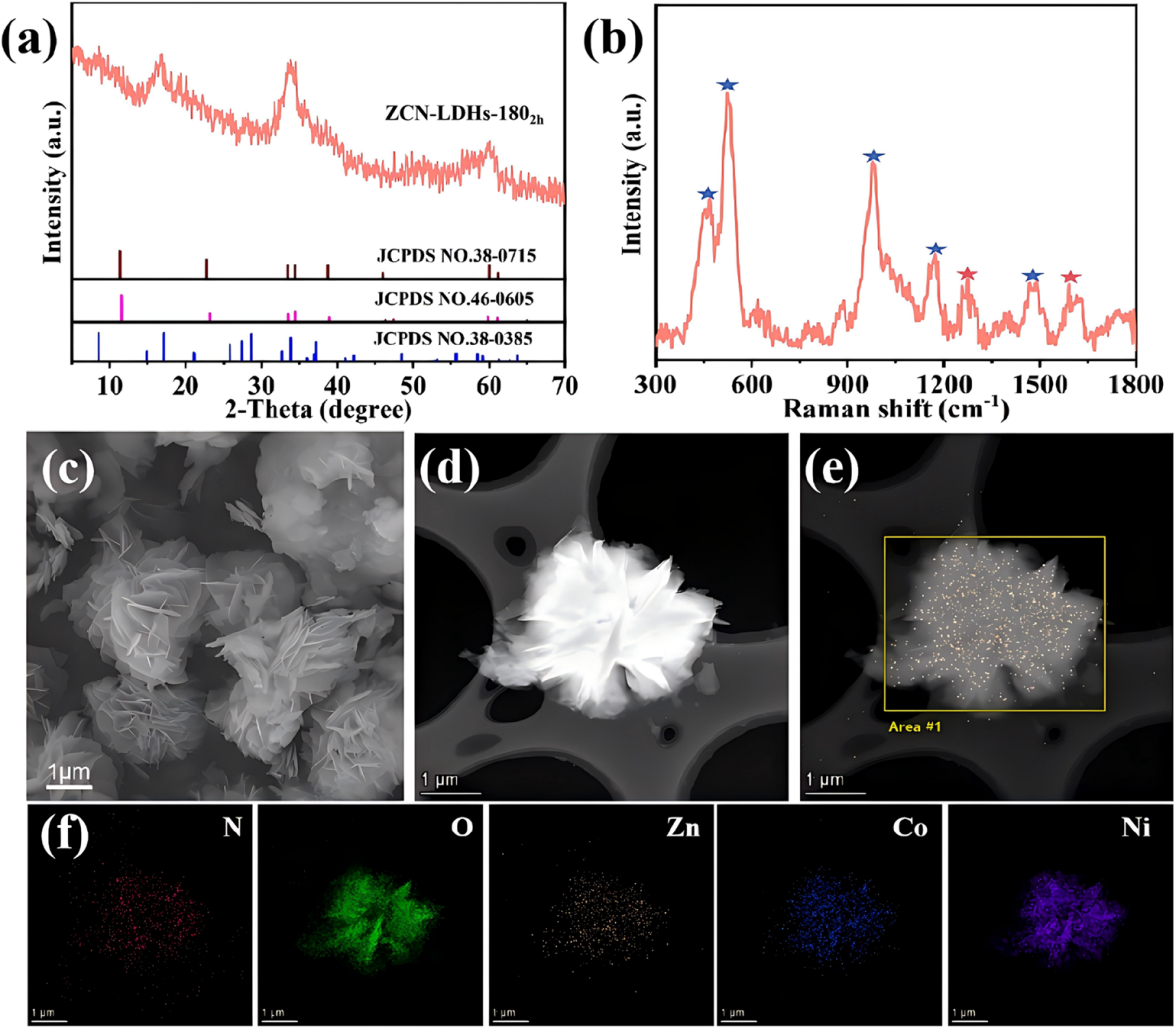
(a) XRD, (b) Raman patterns, (c) SEM, (d) TEM images and (e, f) EDS images of ZCN-LDHs-180_2h_.

As presented in Fig. 1a, the diffraction peaks at 16.8°, 33.9°, and 60.05° correspond to the (002), (006), and (−111) crystal planes of ZCN-LDHs-180_2h_, respectively. To further investigate the structure of ZCN-LDHs-180_2h_, its Raman spectrum was recorded (Fig. 1b). The sample exhibits two prominent peaks at ∼1270 cm^−1^ and 1589 cm^−1^, which are assigned to the D-band and G-band,^30,31^ as also observed in previously reported 2D materials.^32^ The peaks at 456 cm^−1^ and 526 cm^−1^ correspond to Zn–OH/Co–OH/Ni–OH and Zn–O/Co–O/Ni–O,^33^ those at 980 cm^−1^ and 1168 cm^−1^ to SO_4_^2−^ stretching vibrations,^34^ and the peak at 1467 cm^−1^ to C=C.

According to the scanning electron microscopy SEM and TEM images, ZCN-LDHs-180_2h_ exhibits a distinct morphology (Fig. 1c and d): a complex 3D nanoflower architecture self-assembled from numerous ultrathin nanosheets. However, not all Zn/Co/Ni ratios can form this nanoflower-like morphology. Some samples appear as bulk agglomerates without obvious nanoflower features. As shown in Fig. S1, the morphologies of ZCN-LDHs samples with different Zn/Co/Ni ratios vary significantly, and only the optimal ratio (0.2 : 0.2 : 1) forms the most uniform and well-defined nanoflower structure.

Energy-dispersive X-ray spectroscopy (EDS) analysis was performed to determine the elemental content of different samples. Fig. S2a shows the elemental mapping of ZnCo-MOF, clearly revealing a core–shell structure with Zn-MOF as the core and Co-MOF coated on its surface *via in situ* growth. The uniform distribution of C and N verifies the successful synthesis of the bimetallic ZnCo-MOF. In the EDS results of ZCN-LDHs-180_2h_ (Fig. 1e and f), Zn, Co, Ni, O and N elements are clearly detected: Zn and Co are mainly distributed in the inner nanoflower sheets, while Ni is concentrated in the outer ones, verifying the successful synthesis of ZCN-LDHs-180_2h_ with layered nanopetals using ZnCo-MOF as the template. The uniform distribution of C and N indicates that ZCN-LDHs-180_2h_ inherits the C/N-rich property of MOFs. Consistent results from XRD, Raman spectroscopy, and EDS collectively confirm the successful synthesis of ZCN-LDHs-180_2h_.

**Fig. 2:**
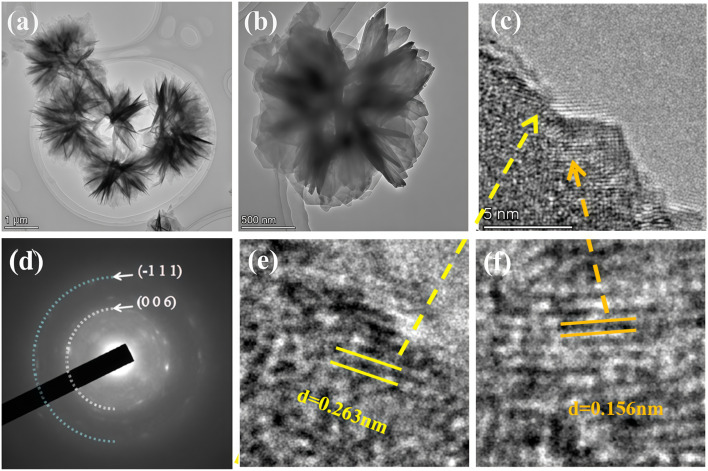
(a–c) TEM images, (d) SAED pattern and (c, e and f) HRTEM images of ZCN-LDHs-180_2h_.

In Fig. 2a–c, high- and low-magnification TEM images show that the material is composed of ultrathin nanosheets self-assembled into a 3D nanoflower structure with large interlayer spacing, consistent with the above analysis. Fig. 2d presents the SAED pattern of the sample. The rings consisting of bright spots indicate the polycrystalline nature of ZCN-LDHs-180_2h_, and the two diffraction rings correspond to the (−111) and (006) crystal planes of the material, which is in good agreement with the XRD results. As shown in Fig. 2e and f, two sets of lattice fringes with interplanar spacings of 0.263 nm and 0.156 nm are observed in the HRTEM image, corresponding to the (−111) and (006) crystal planes of ZCN-LDHs-180_2h_, respectively.

**Fig. 3:**
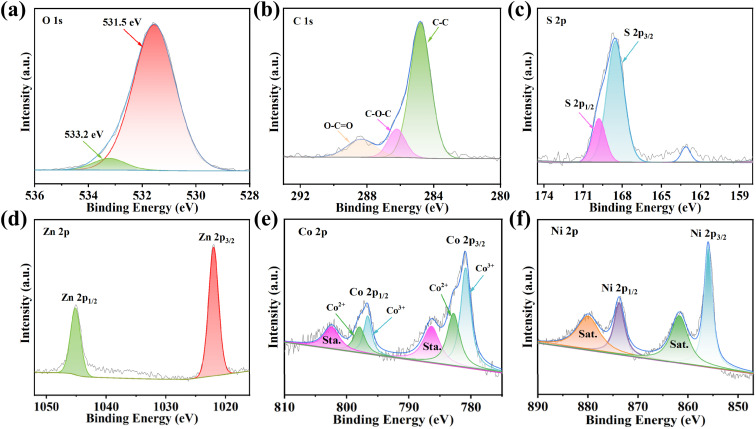
The high-resolution XPS survey of (a) O 1s, (b) C 1s, (c) S 2p, (d) Zn 2p, (e) Co 2p and (f) Ni 2p of ZCN-LDHs-180_2h_.

X-ray photoelectron spectroscopy (XPS) was employed to analyze the valence states of elements and possible substance components in ZCN-LDHs-180_2h_. Fig. 3 shows high-resolution XPS spectra of (a) O 1s, (b) C 1s, (c) S 2p, (d) Zn 2p, and (e) Co 2p of ZCN-LDHs-180_2h_. In Fig. 3a, the O 1s spectrum exhibits two main peaks at 531.6 eV and 533.2 eV, which are characteristic of hydroxyl groups and interlayer water molecules in LDH materials, consistent with previous reports.^35–37^Fig. 3b (high-resolution C 1s spectrum) shows characteristic peaks at 284.4, 286.2, and 288.5 eV, attributed to C signal peaks in C–C, C–O–C, and O–C=O bonds, respectively.^38^Fig. 3c (high-resolution S 2p spectrum) presents characteristic peaks at 168.6 eV and 169.8 eV, corresponding to S 2p_3/2_ and S 2p_1/2_ (both belong to S signal peaks in the SO_4_^2−^ group),^38^ consistent with Raman spectrum analysis of ZCN-LDHs-180_2h_. Fig. 3d (high-resolution Zn 2p spectrum) shows two characteristic peaks at 1021.9 eV and 1045.2 eV, corresponding to Zn 2p_3/2_ and Zn 2p_1/2_, respectively, confirming the presence of Zn^2+^.^39^ Based on the binding energy positions, Zn^2+^ likely acts as a structural stabilizer within the LDH layers. Fig. 3e (high-resolution Co 2p spectrum) exhibits two prominent spin–orbit peaks by their corresponding satellite peaks, with binding energies consistent with the reported standard values of Co 2p. The peaks at 780.8 eV and 796.6 eV are attributed to Co^3+^; those at 782.8 eV and 798 eV to Co^2+^, and the peaks at 786.4 eV and 802.5 eV to the satellite peaks Co 2p.^40,41^Fig. 3f (high-resolution Ni 2p spectrum) also shows two prominent spin–orbit peaks and corresponding satellite peaks, with binding energies consistent with reported standard values of Ni 2p. The relatively strong peaks at 855.8 eV and 873.8 eV are assigned to Ni 2p_3/2_ and Ni 2p_1/2_, respectively, while those at 861.7 eV and 879.9 eV are ascribed to the Ni 2p satellite peaks.^40,41^ Based on the above analysis, ZCN-LDHs-180_2h_ with a hierarchical structure and nanosheet-petal morphology was successfully prepared by a solvothermal method at 180 °C for 2 h, using ZnCo-MOF as a precursor with *in situ* introduction of Ni ions.

Reaction temperature and duration critically influence nanomaterial synthesis, leading to significant variations in morphology, size, crystallinity, and phase composition. As shown in Fig. S2b, the XRD pattern of the synthesized ZnCo-MOF matches perfectly with previous reports,^42^ confirming successful precursor preparation. As shown in Fig. S2c, the samples synthesized at 140–170 °C retain the crystalline phase of precursor, with consistent diffraction peaks. A phase transition occurs at 180 °C, leading to the formation of ZCN-LDHs-180_2h_. At 190 °C, diffraction peak at 16.8° disappears, which can be attributed to the strengthened interactions between interlayer anions (*e.g.*, SO_4_^2−^, OH^−^) and LDHs layers, thus inducing lattice contraction and decreased interlayer spacing.^43–45^ These results indicate that 180 °C is the optimal temperature for the synthesis of ZCN-LDHs-180_2h_.

SEM analysis reveals morphological evolution with temperature (Fig. S3a–h). Zn-MOF and Co-MOF exhibit regular dodecahedral structures (Fig. S3a and b), while ZnCo-MOF maintains this morphology *via* the *in situ* growth of Co-MOF on Zn-MOF (Fig. S3c).^46^ ZCN-LDHs-140 shows rough block-like structures (Fig. S3d), whereas ZCN-LDHs-160 presents block-like cores with dense surface nanosheets (Fig. S3e). At 170 °C, the nanosheets grow larger but remain irregularly stacked (Fig. S3f). ZCN-LDHs-180 forms well-defined 3D nanoflower architectures through the ordered nanosheet self-assembly (Fig. S3g), while 190 °C leads to structural collapse and severe agglomeration (Fig. S3h). Therefore, 180 °C yields the optimal morphology.

XRD patterns (Fig. S4) show that the characteristic peaks of the MOF precursor are preserved after 0.5 h and 1 h reaction. Extending the reaction time to 2 h at 180 °C induces a phase transition to ZCN-LDHs-180_2h_. For the sample obtained after 3 h, the low-angle diffraction peak shifts to lower angles, indicating interlayer expansion due to the rearrangement of intercalated anions (SO_4_^2−^, OH^−^).^47–49^ SEM analysis (Fig. S5a–d) demonstrates a time-dependent morphological evolution: prolonged reaction duration promotes the ordered growth of nanosheets and their self-assembly into nanoflower structures. However, an excessive reaction time of 3 h causes uncontrolled nanosheet overgrowth, resulting in disordered stacking and disruption of the 3D architecture. These structural and morphological features suggest that the 180 °C, 2 h condition yields optimally arranged nanosheets with enlarged interlayer spacing. This architecture facilitates OH^−^ ion transport during charge–discharge cycles, thereby enhancing charge storage, energy density, and specific capacitance.

To further analyze the microstructure and specific surface area of the composite materials, we employed N_2_ adsorption–desorption techniques to examine Zn-MOF, Co-MOF, ZnCo-MOF and ZCN-LDH-180_2h_. As shown in Fig. S6, the isotherms of all samples exhibited Type IV characteristics, indicating that all three materials possess mesoporous structures.^50^ BET test calculations revealed that ZCN-LDH-180_2h_ exhibited significantly higher specific surface area (2249.64 m^2^ g^−1^) compared to Zn-MOF (1743.63 m^2^ g^−1^), Co-MOF (2100.37 m^2^ g^−1^) and ZnCo-MOF (1880.26 m^2^ g^−1^). This increase in specific surface area originates from the structural transformation from the compact MOF precursors to the 3D nanoflower architecture assembled from ultrathin nanosheets. Such a hierarchical structure effectively inhibits nanosheet stacking, exposes more active sites, and facilitates electrolyte ion transport, which is consistent with previous reports on MOF-derived materials.^51^

### 3.2 Comparative study of binary and ternary LDHs

To evaluate ternary LDH advantages, three binary systems were synthesized: ZC-LDHs and ZN-LDHs (from Zn-MOF with *in situ* Co^2+^/Ni^2+^ introduction), and CN-LDHs (from Co-MOF with *in situ* Ni^2+^ introduction). Fig. S7 presents the XRD patterns of the three binary LDH samples. The diffraction peaks of ZC-LDHs, ZN-LDHs and CN-LDHs can be indexed to the standard reference patterns of Zn(OH)_2_ (PDF#38-0308), Co(OH)_2_ (PDF#46-0605) and Ni(OH)_2_ (PDF#38-0715), respectively, confirming the formation of phase-pure binary LDHs.

ZC-LDHs exhibits a microscale spherical structure constructed by interconnected nanosheets (Fig. S8a and b), however, the nanosheets are poorly dispersed and severely agglomerated, which limits the exposure of active sites. ZN-LDHs (Fig. S8c and d) show a rough surface with abundant fine concave-convex features, attributed to the regulatory effect of metal ions on crystal growth. CN-LDHs appear as nanoscale spherical particles with smooth surfaces (Fig. S8e and f). This morphology corresponds to a relatively small specific surface area and a lack of well-defined porous structures, thereby reducing the number of surface reactive sites. Collectively, these observations highlight that Zn^2+^ plays a critical role in modulating LDH crystal growth and porous structure formation. Furthermore, the combination of Zn^2+^ and Co^2+^ is hypothesized to induce a synergistic effect that further facilitates pore formation and expansion—an important structural advantage for electrochemical applications.

In marked contrast to binary LDHs, ZCN-LDHs-180_2h_ exhibits a complex 3D nanoflower architecture self-assembled from numerous ultrathin nanosheets. These nanosheets interlace and curl to form a stable 3D network, which mitigates stacking and enhances structural stability. Such structural features are crucial for sustaining long-term repeated charge–discharge cycling in supercapacitor applications.

### 3.3 Electrochemical performance

To evaluate the electrochemical performance of the products under different reaction conditions, the samples synthesized at 140–190 °C were characterized by CV, GCD, and EIS. As shown in Fig. S9a (CV curves at 2 mV s^−1^), the samples prepared below 180 °C exhibit two oxidation peaks, which gradually shift to higher potentials and coalesce as the temperature increases. At 180 °C, these peaks integrate into a single characteristic peak for ZCN-LDHs-180_2h_, this phenomenon suggests a strong electronic coupling among the metal ions, which could be interpreted as a manifestation of the synergistic interaction between Zn^2+^, Co^2+^, and Ni^2+^, potentially leading to a more uniform redox behavior of the material.^28^ The GCD profiles at 1 A g^−1^ (Fig. S9b) reveal that ZCN-LDHs-180_2h_ displays the longest discharge time, delivering a specific capacitance of 2288 F g^−1^, superior to that of ZCN-LDHs-140/160/170/190. As illustrated in Fig. S9c, ZCN-LDHs-180_2h_ maintains high capacitance even at large current densities, outperforming most reported hydroxide electrodes. EIS analysis (Fig. S9d) shows that ZCN-LDHs-180_2h_ exhibit the steepest slope in the low-frequency region, which is characteristic of a lower diffusion resistance and thus implies faster ion transport.^52^ This enhanced kinetic behavior is plausibly facilitated by the material's unique 3D nanoflower architecture, which provides abundant and accessible pathways for electrolyte ions. Accordingly, 180 °C was identified as the optimal synthesis temperature for subsequent investigation of reaction time.

Fig. S10 presents the electrochemical performance of samples synthesized at 180 °C for 0.5–3 h. The CV curves at 2 mV s^−1^ (Fig. S10a) show that the samples obtained after 0.5 h and 1 h exhibit two distinct oxidation peaks, indicative of an unstable or incompletely formed structure. As the reaction proceeds to 2 h, these peaks merge into a single oxidation peak at the characteristic potential of ZCN-LDHs-180_2h_, indicating structural optimization and ion rearrangement. GCD profiles at 1 A g^−1^ (Fig. S10b) show that ZCN-LDHs-180_2h_ achieve the highest specific capacitance (2288 F g^−1^), outperforming the other samples. Rate capability tests (Fig. S10c) demonstrate superior capacity retention for ZCN-LDHs-180_2h_, confirming its excellent rate performance. EIS analysis (Fig. S10d) further shows the steepest low-frequency slope for ZCN-LDHs-180_2h_, consistent with enhanced ion diffusion and electron transfer due to metal ion synergy. Accordingly, the sample synthesized at 180 °C for 2 h was selected as the optimal sample for subsequent experimental studies.

To determine the optimal Zn/Co/Ni ratio, twelve ZCN-LDHs samples with different metal molar ratios (summarized in Experimental Section 2.2) were synthesized and evaluated by CV and GCD measurements. As shown in Fig. S11 and S12, the sample with a Zn : Co : Ni ratio of 0.2 : 0.2 : 1 exhibited the highest specific capacitance (2288 F g^−1^ at 1 A g^−1^) and the best rate performance among all tested ratios. Therefore, this optimal composition was selected for subsequent characterizations and device assembly.

**Fig. 4:**
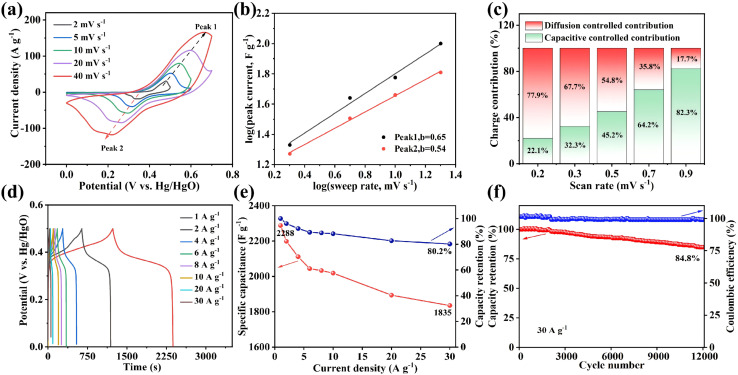
ZCN-LDHs-180_2h_ sample: (a) CV curves at different scan rates, (b) plots of log(*i*) *vs.* log(*v*) at different peak currents, (c) capacitance contribution plots at different scan rates, (d) GCD curves at different current densities, (e) specific capacitance plots at different current densities and (f) cycling stability plot.

Fig. 4 presents the electrochemical properties of ZCN-LDHs-180_2h_. The CV curves in Fig. 4a show two distinct redox peaks, confirming the pseudocapacitive nature of the electrode material. In contrast to ideal electric double-layer capacitance, pseudocapacitance originates from reversible faradaic reactions occurring at redox-active sites on or near the electrode surface. The detailed faradaic reactions involved in the ZCN-LDHs-180_2h_ electrode have been reported in previous studies.^53–56^ The CV curves retain excellent shape consistency at various scan rates, demonstrating superior rate capability. As the scan rate increases, the anodic peak (Peak 1) shifts toward more positive potentials, while the cathodic peak (Peak 2) shifts toward more negative potentials. This peak shift is attributed to a transition in the electrochemical reaction kinetics from diffusion-controlled to surface-controlled processes.

To investigate the electrode reaction kinetics, the relationship between peak current (*i*) and scan rate (*ν*) was analyzed according to the power law: *i*(*ν*) = *aν*^*b*^, where *b* is determined from the slope of the log(*i*) *versus* log(*ν*) plot (Fig. 4b).^57^ A *b*-value of 0.5 is characteristic of a diffusion-controlled process, whereas *b* = 1 corresponds to a surface-controlled capacitive mechanism. Calculations yield *b*-value of 0.65 for the oxidation peak and 0.54 for the reduction peak, indicating that the charge storage mechanism is predominantly diffusion-controlled, with a substantial contribution from surface capacitive effects.^58^Fig. 4c presents the capacitance contribution at various scan rates: at low scan rates, the reaction is governed by diffusion-limited OH^−^ transport; as the scan rate increases, OH^−^ transfer at the electrode surface accelerates, and the surface capacitive contribution becomes increasingly prominent.

As shown in Fig. 4d and e, ZCN-LDHs-180_2h_ delivers a mass-specific capacitance of 2288 F g^−1^ at 1 A g^−1^, and still maintains 1835 F g^−1^ (80.2% capacitance retention) at 30 A g^−1^, manifesting superior rate capability for rapid energy storage and release during fast charge–discharge processes. Fig. 4f shows long-cycle stability: at 30 A g^−1^, the capacitance retention remains 84.8% after 12 000 consecutive charge–discharge cycles, revealing exceptional long-term cycling stability with negligible capacitance decay. A performance comparison between this work and previously reported studies is summarized in Table S1, which further validates the outstanding electrochemical properties of ZCN-LDHs-180_2h_.

To elucidate the superior electrochemical performance of ternary LDHs, ZCN-LDHs-180_2h_ was electrochemically characterized alongside three binary reference samples (ZC-LDHs, ZN-LDHs, CN-LDHs) under identical testing conditions. As illustrated in Fig. S13a, all four materials exhibit distinct redox peaks, confirming reversible redox reactions. ZC-LDHs displays two separate oxidation peaks corresponding to the different redox potentials of Zn^2+^ and Co^2+^/Co^3+^, whereas ZN-LDHs, CN-LDHs, and ZCN-LDHs-180_2h_ (containing Ni^2+^) exhibit a single merged oxidation peak. This can be attributed to the strong interfacial interactions and synergistic effects among the metal ions, which induce shifts in redox potentials and lead to peak overlap. Notably, ZCN-LDHs-180_2h_ exhibits the largest integrated area of the CV curve, suggesting more efficient charge transport and storage, which is favored by its unique 3D nanoflower architecture with a larger specific surface area.

Fig. S13b presents the GCD profiles at 1 A g^−1^. ZCN-LDHs-180_2h_ exhibits the longest discharge duration, delivering a mass-specific capacitance of 2288 F g^−1^, which is significantly higher than ZC-LDHs (711 F g^−1^), ZN-LDHs (938.6 F g^−1^), and CN-LDHs (1944.8 F g^−1^). Fig. S13c (plot of specific capacitance *versus* current density) shows ZCN-LDHs-180_2h_ retains substantially higher capacitance than its binary counterparts even at a high current density of 30 A g^−1^. This superior rate performance, when combined with the EIS results (Fig. S13d) showing the lowest contact resistance (0.6 Ω) and steepest diffusion slope for ZCN-LDHs-180_2h_, strongly suggests that the ternary composition synergistically enhances both charge transfer and ion diffusion kinetics within the electrode material. Notably, the steepest low-frequency slope of ZCN-LDHs-180_2h_ is attributed to a dual contribution: (ⅰ) its unique 3D nanoflower architecture, which maximizes the electrode–electrolyte contact area; and (ii) a hypothesized synergistic effect among Zn^2+^, Co^2+^, Ni^2+^, which may promote more efficient ion exchange and charge transfer, thereby reinforcing the structural stability and contributing to the reduced impedance.

**Fig. 5:**
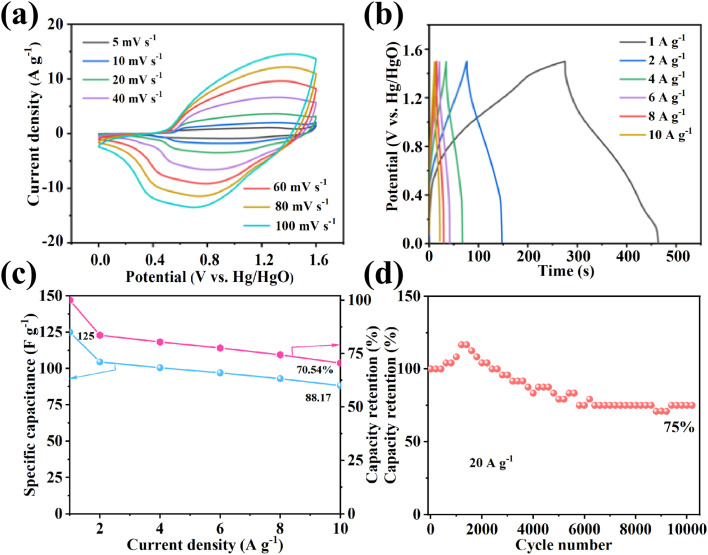
ZCN-LDHs-180_2h_//AC: (a) CV curves at different scan rates, (b) GCD curves at different current densities, (c) specific capacitance plots at different current densities and (d) cycle stability curve of ZCN-LDHs-180_2h_//AC.

An asymmetric supercapacitor (ASC) was constructed using ZCN-LDHs-180_2h_ as the positive electrode and commercial AC as the negative electrode. Fig. 5a presents CV curves at scan rates ranging from 5 to 100 mV s^−1^ within a voltage window of 0–1.5 V: the CV curves maintain a well-defined rectangular shape without significant distortion, confirming excellent rate performance.

In GCD curves (Fig. 5b), distinct charge–discharge plateaus are observed, which correspond to the redox peaks in the CV curves, revalidating the pseudocapacitive behavior. The GCD curves exhibit good symmetry, indicating high coulombic efficiency and excellent structural stability. At a current density of 1 A g^−1^, the specific capacitance of the ASC device reaches 125 F g^−1^. Fig. 5c shows that the specific capacitance decreases gradually with the increase of current density: at 10 A g^−1^, the device delivers a specific capacitance of 88.17 F g^−1^ with a capacitance retention of 70.54%, demonstrating outstanding rate performance. The cycling stability curve (Fig. 5d) reveals a gradual capacity decay upon prolonged cycling, which can be attributed to the Jahn–Teller distortion of Ni^3+^, partial dissolution of transition metal ions in the alkaline electrolyte, and gradual weakening of the electrode interface, as commonly reported for LDH-based materials.^59^ Nevertheless, the 84.8% capacitance retention after 12 000 cycles at 30 A g^−1^ remains competitive, demonstrating the structural robustness of the 3D nanoflower architecture.

Fig. S14 presents the Ragone plot (energy density *vs.* power density) of the ASC device: the energy density decreases gradually with the increase of power density, which is attributed to incomplete charge storage at high current densities. The device exhibits a maximum energy density of 39.1 Wh kg^−1^ at 750 W kg^−1^; even at 7.5 kW kg^−1^, it still maintains 27.6 Wh kg^−1^, outperforming most previously reported devices (Table S2).

## 4. Conclusions

In this study, a ternary ZnCoNi-LDHs material (denoted as ZCN-LDHs-180_2h_) with a unique three-dimensional nanoflower architecture was successfully synthesized. The material was prepared using ZnCo-MOF as a precursor template, into which Ni^2+^ introduced *in situ*, followed by an optimized solvothermal treatment at 180 °C for 2 h. This strategy leverages the structural-directing role of the MOF template, offering a facile route to high-performance ternary LDHs. As a supercapacitor electrode material, the ZCN-LDHs-180_2h_ demonstrates exceptional electrochemical performance, delivering a high specific capacitance of 2288 F g^−1^ at a current density of 1 A g^−1^. Furthermore, it retains a capacitance of 1835 F g^−1^ even at a high current density of 30 A g^−1^, corresponding to an 80.2% retention rate, which demonstrates its excellent rate capability.

The remarkable performance of the ZCN-LDHs-180_2h_ electrode can be rationalized by its advantageous structural and compositional features. The experimental evidence suggests that the 3D hierarchical nanoflower architecture plays a crucial role by effectively preventing nanosheet stacking, which in turn exposes a greater number of electroactive sites and shortens ion diffusion pathways. Furthermore, the results from electrochemical analysis and material characterization point towards a beneficial synergistic interaction among the Zn, Co, and Ni ions. This synergy is proposed to modulate the material's electronic structure, thereby enhancing its intrinsic redox activity and contributing to the observed structural stability during prolonged cycling. Collectively, these factors are believed to underpin the high specific capacitance, excellent rate capability, and superior cycling stability achieved in this work.

## Author contributions

Gaofu Liu: conceptualization, methodology, validation, investigation, writing-original draft. Kunyu Hao: conceptualization, validation, investigation, writing-original draft. Zhuanyu Liu: validation, investigation, resources, data curation. Yiwen Tang: conceptualization, supervision, funding acquisition, writing-review & editing. Yonggang Wu: supervision, writing-review & editing.

## Conflicts of interest

There are no conflicts of interest to declare.

## Supplementary Material

RA-016-D5RA09868H-s001

## Data Availability

The data supporting this article have been included as part of the supplementary information (SI). Supplementary information is available. See DOI: https://doi.org/10.1039/d5ra09868h.
