# Supplementary material for: MOF-derived ternary ZnCo–Ni LDHs for high-energy-density supercapacitors: synergistic effects and enhanced ion transfer
Source: RSC Adv. 2026 Apr 22;16(23):21256–65. doi: 10.1039/d5ra09868h (PMC13102158; doi:10.1039/d5ra09868h)
Supplement: RA-016-D5RA09868H-s001 [file RA-016-D5RA09868H-s001.pdf]

Supplementary Information (SI) for RSC Advances.
This journal is © The Royal Society of Chemistry 2026

Supporting Information

MOF-Derived Ternary ZnCo-Ni LDHs for High-Energy-Density Supercapacitors: Synergistic Effects and Enhanced Ion Transfer

Gaofu Liu[a,b,1], Kunyu Hao[b,1], Zhuanyu Liu[b], Yiwen Tang*[b], Yonggang Wu*[a]

a School of Physics and Electronic Science, Guizhou Education University, Guiyang 550018, P. R. China

b Institute of Nano-Science and Technology, College of Physical Science and Technology, Central China Normal University, Wuhan, 430079, China

*Corresponding author: Yiwen Tang, Tel: +86-27-67867947; Fax: +86-27-67861185; e-mail: ywtang@ccnu.edu.cn

*Corresponding author: Yonggang Wu, e-mail: ygwu0946@163.com

[1]These authors contribute equally to this work.

1. Sample Characterization

Sample characterization methods: An Empyrean-type X-ray diffractometer (Cu Kα line $\lambda = 1.5418$ Å) was used to obtain the XRD pattern, which was compared with the standard cards to determine the phase. The morphology and structure of the samples were characterized by field emission scanning electron microscope (SEM) (JSM-6700F, JEOL) and transmission electron microscope (TEM) (JEM-2010FEF, JEOL). The elemental content of the samples was analyzed by energy-dispersive spectrometer (EDS). A VG Multilab 2000 type X-ray photoelectron spectrometer (XPS) was used to determine the chemical components and element valence states on the sample surface, and the characteristic peak of C 1s at 284.6 eV was used as a reference for calibrating the binding energies of the elements. A Raman spectrometer (laser wavelength of 532 nm) was used to obtain the Raman spectrum of the sample.

2 Electrochemical Testing

Electrochemical testing methods: The CHI760 electrochemical workstation was used to conduct electrochemical tests on the samples. ZCN-LDHs-180_{2h} was used as the working electrode, a platinum sheet as the counter electrode, and Hg/HgO as the reference electrode. In a 2 M KOH aqueous solution at room temperature, three-electrode electrochemical tests of cyclic voltammetry (CV), galvanostatic charge-discharge (GCD), electrochemical impedance (EIS, frequency range of 0.01 Hz - 10 kHz), and cycling stability were carried out within a voltage range of 0 to 0.6 V.

The specific capacitance, energy density, and power density in the three-electrode system and the assembled asymmetric supercapacitor were calculated by the following formulas:

$$C_{cv} = (\int i dV)/(v \times m \times \Delta V) \quad (1)$$

C_{cv} is the specific capacitance calculation based on the CV curve, where $\int i dV$ is the integral area of the CV curve, v is the scan rate, m is the mass of the active material, and ΔV is the working-potential range.

$$C_{GCD} = (I \times \delta t)/(m \times \Delta V) \quad (2)$$

C_{GCD} is the specific capacitance calculation based on the GCD curve, where I is the discharge current (A), m is the mass of the active material (g), δt is the discharge time, and ΔV is the working-potential range.

When assembling a supercapacitor, first, according to the charge balance theory, the loading amounts of the positive and negative active materials were matched based on their specific capacitance and voltage ranges. The specific calculation is as follows:

$$m_{+} \times C_{+} \times \Delta V_{+} = m_{-} \times C_{-} \times \Delta V_{-} \quad (3)$$

m_{+} and m_{-} are the loadings of the positive and negative electrode active materials, respectively, and C_{+}, C_{-}, ΔV_{+}, and ΔV_{-} are the specific capacitances and voltage windows of the positive and negative electrodes, respectively.

$$E=0.5C\times\Delta V^{2} \quad (4)$$

$$P=E/\Delta t \quad (5)$$

C represents the specific capacitance of the electrode material (F g^{-1}), ΔV is the working potential range, E is the energy density, P is the power density, and Δt is the discharge time.

3 Supporting figures and tables

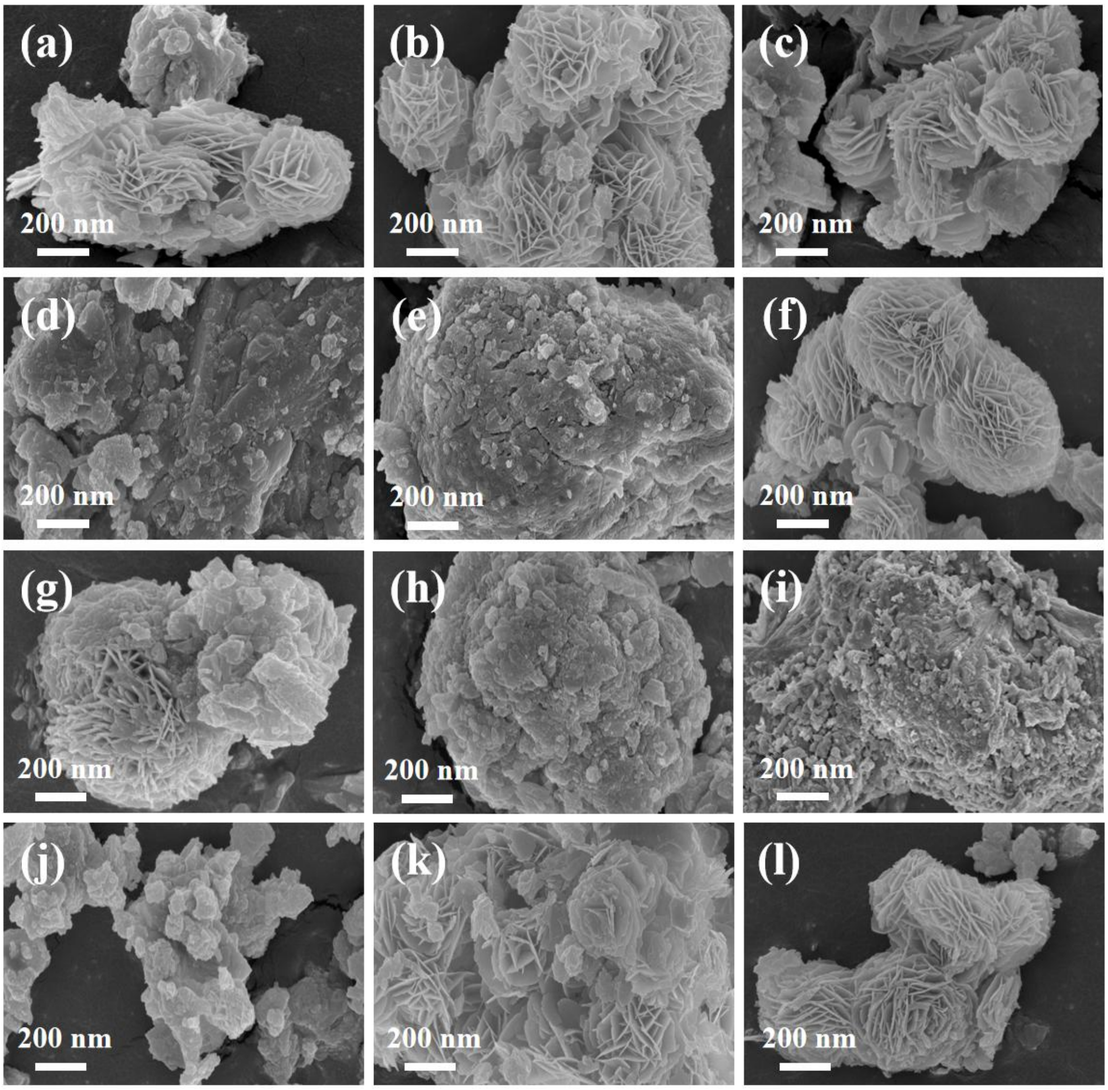

Fig. S1 SEM images of all ZCN-LDHs samples with different Zn/Co/Ni ratios: (a) ZCN-LDHs$_1$-180$_{2h}$, (b) ZCN-LDHs-180$_{2h}$, (c) ZCN-LDHs$_2$-180$_{2h}$, (d) ZCN-LDHs$_3$-180$_{2h}$, (e) ZCN-LDHs$_4$-180$_{2h}$, (f) ZCN-LDHs$_5$-180$_{2h}$, (g) ZCN-LDHs$_6$-180$_{2h}$, (h) ZCN-LDHs$_7$-180$_{2h}$, (i) ZCN-LDHs$_8$-180$_{2h}$, (j) ZCN-LDHs$_9$-180$_{2h}$, (k) ZCN-LDHs$_{10}$-180$_{2h}$ and (l) ZCN-LDHs$_{11}$-180$_{2h}$.

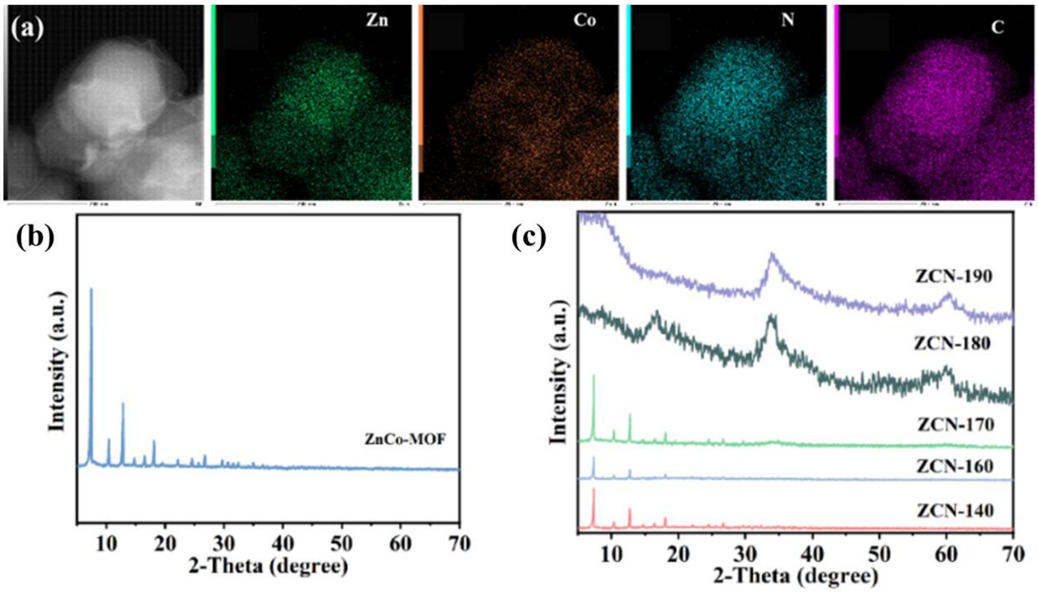

Fig. S2 (a) EDS images, (b) XRD pattern of ZnCo-MOF precursor and (c) XRD patterns of products at different temperatures of 140, 160, 170, 180, and 190 °C via solvothermal method.

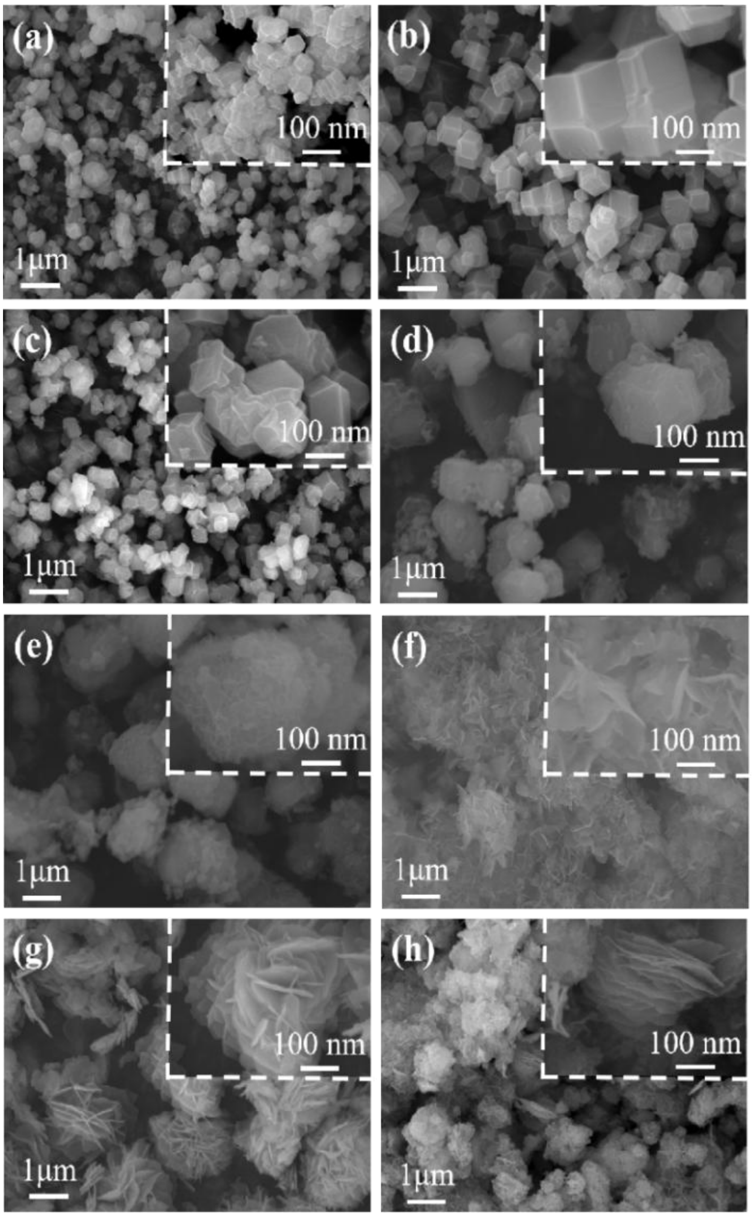

Fig. S3 SEM images of samples (a) Zn-MOF, (b) Co-MOF, (c) ZnCo-MOF, (d) ZCN-LDHs-140, (e) ZCN-LDHs-160, (f) ZCN-LDHs-170, (g) ZCN-LDHs-180 and (h) ZCN-LDHs-190.

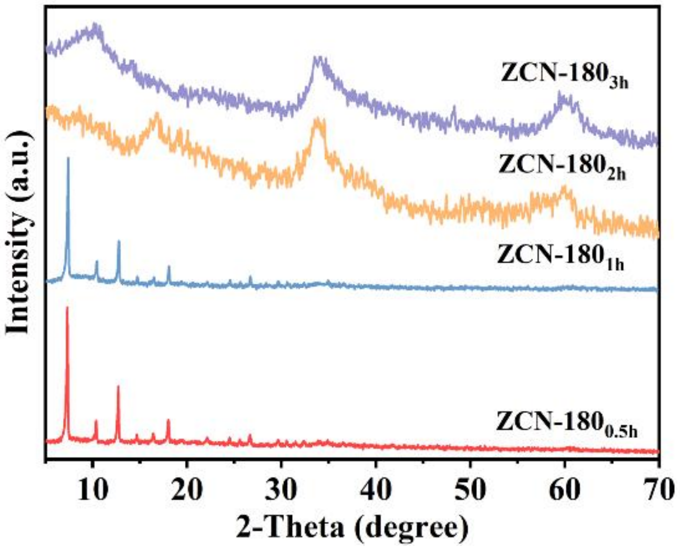

Fig. S4 XRD patterns of products at 180 °C for different reaction times of 0.5 h, 1 h, 2 h, and 3 h.

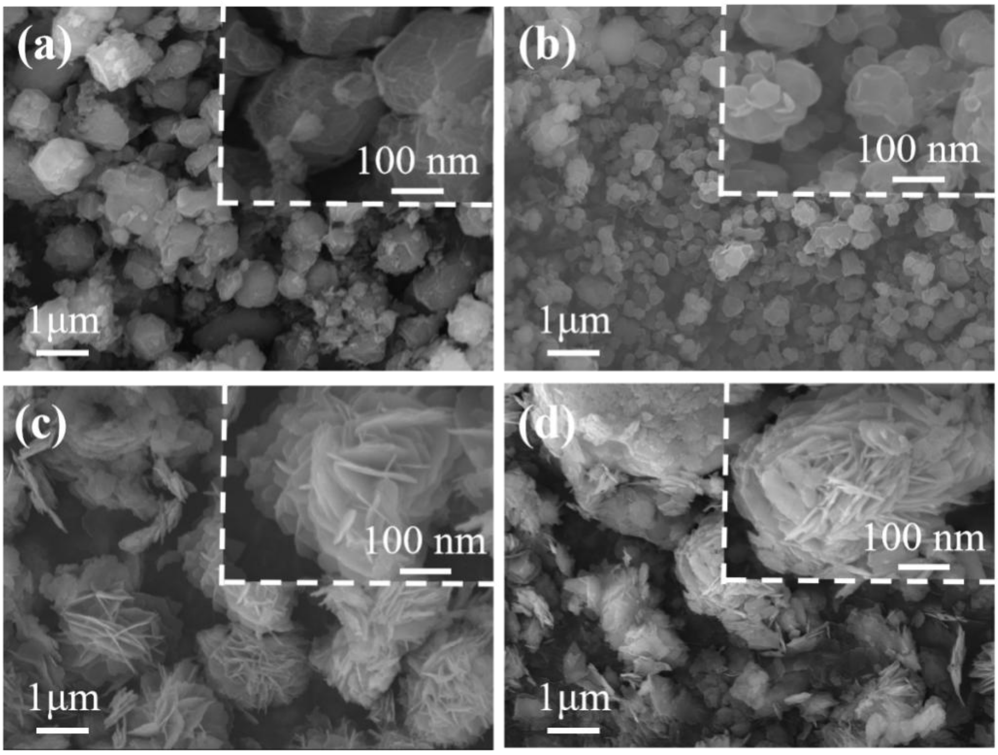

Fig. S5 SEM images of (a) ZCN-LDHs-$180_{0.5h}$, (b) ZCN-LDHs-180_{1h}, (c) ZCN-LDHs-180_{2h} and (d) ZCN-LDHs-180_{3h}.

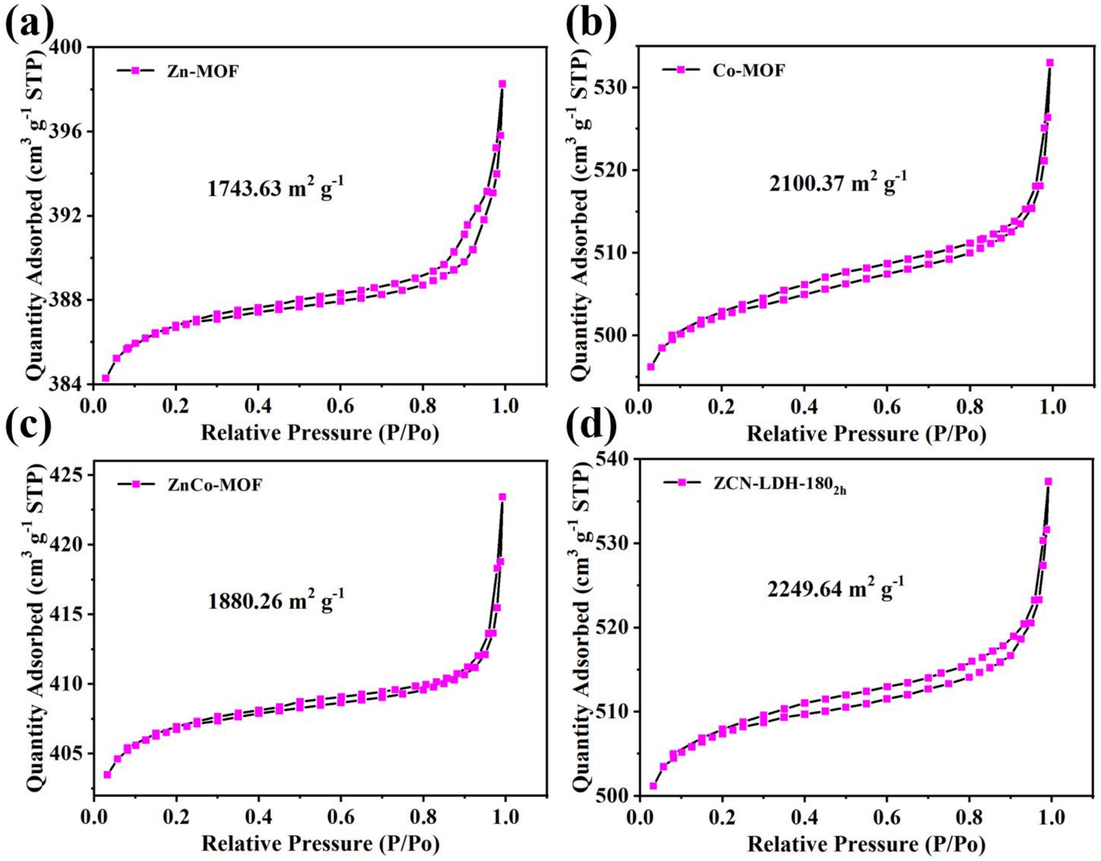

Fig. S6 N_2 adsorption-desorption isotherms of (a) Zn-MOF, (b) Co-MOF, (c) ZnCo-MOF and (d) ZCN-LDH-180_{2h}.

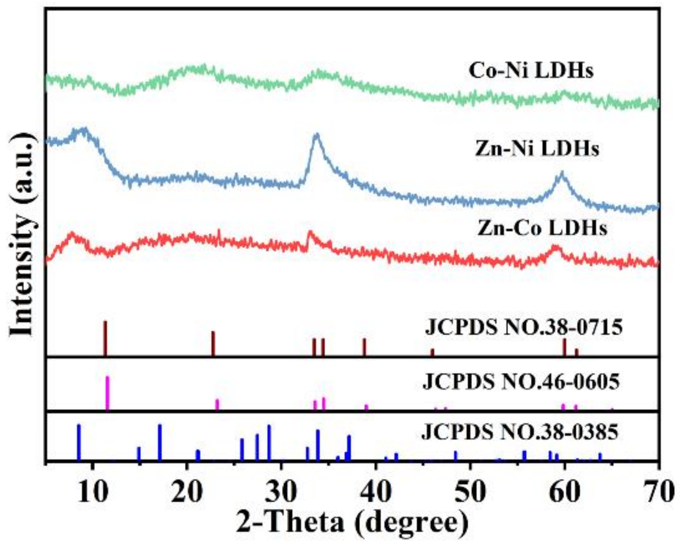

Fig. S7 XRD patterns of ZC-LDHs, ZN-LDHs and CN-LDHs.

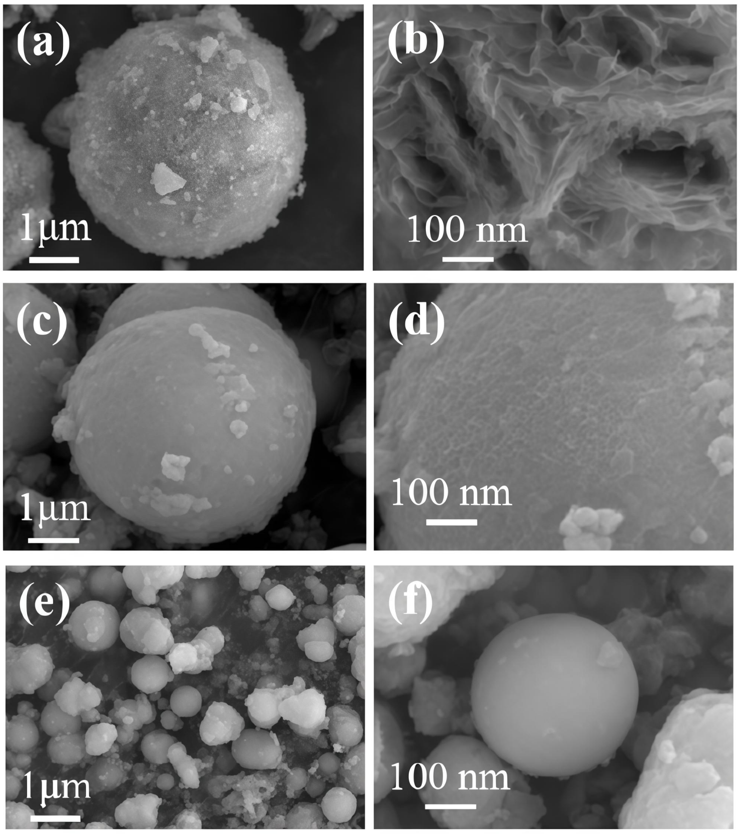

Fig. S8 SEM images of (a, b) Zn-Co LDHs, (c, d) Zn-Ni LDHs and (e, f) Co-Ni LDHs.

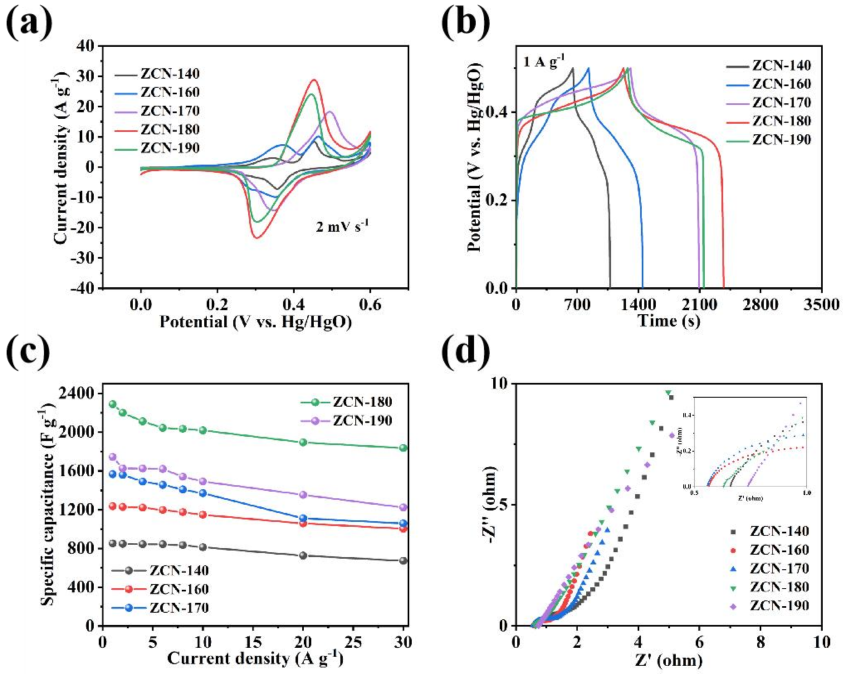

Fig. S9 Samples prepared at different temperatures (140, 160, 170, 180, and 190 °C): (a) CV curves at a scan rate of 2 mV s^{-1}, (b) GCD curves at a current density of 1 A g^{-1}, (c) Specific capacitance plots at different current densities and (d) EIS plots.

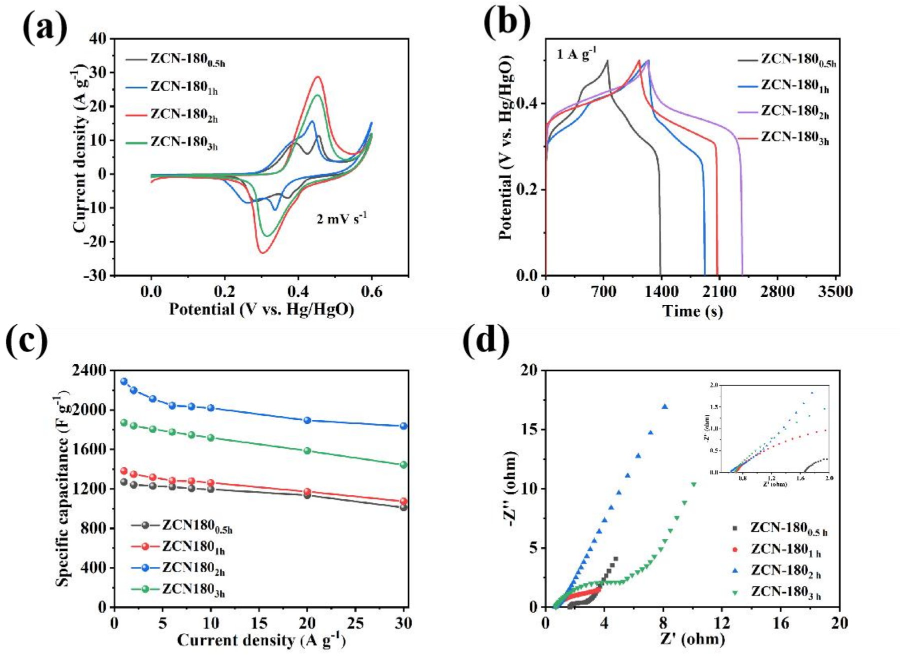

Fig. S10 Samples from solvothermal reaction at 180 °C with different durations (0.5 h, 1 h, 2 h, 3 h): (a) CV curves at 2 mV s^{-1}, (b) GCD curves at 1 A g^{-1}, (c) Specific capacitance plots at different current densities and (d) EIS plots.

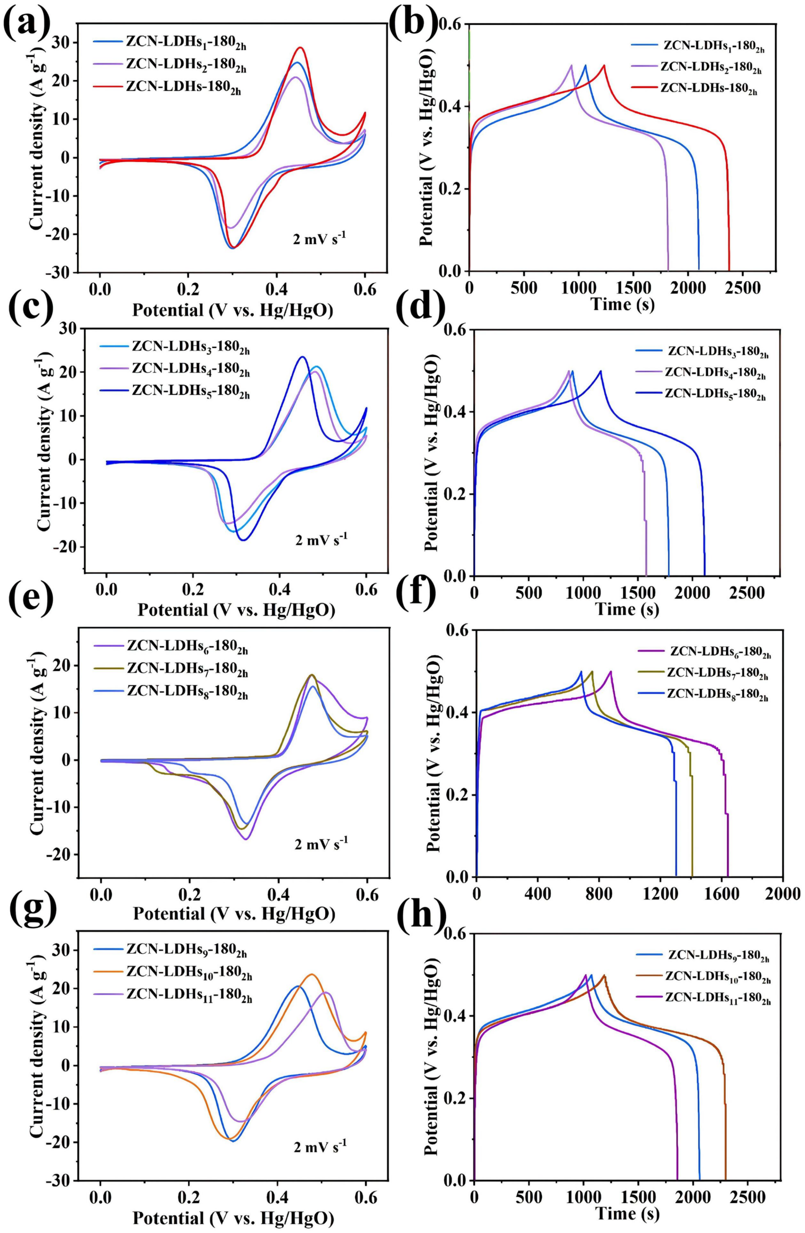

Fig. S11 Electrochemical performance of ZCN-LDHs samples with different component ratios: (a, c, e, and g) CV curves measured at a scan rate of 2 mV s^{-1}, and (b, d, f, and h) GCD curves tested at a current density of 1 A g^{-1}.

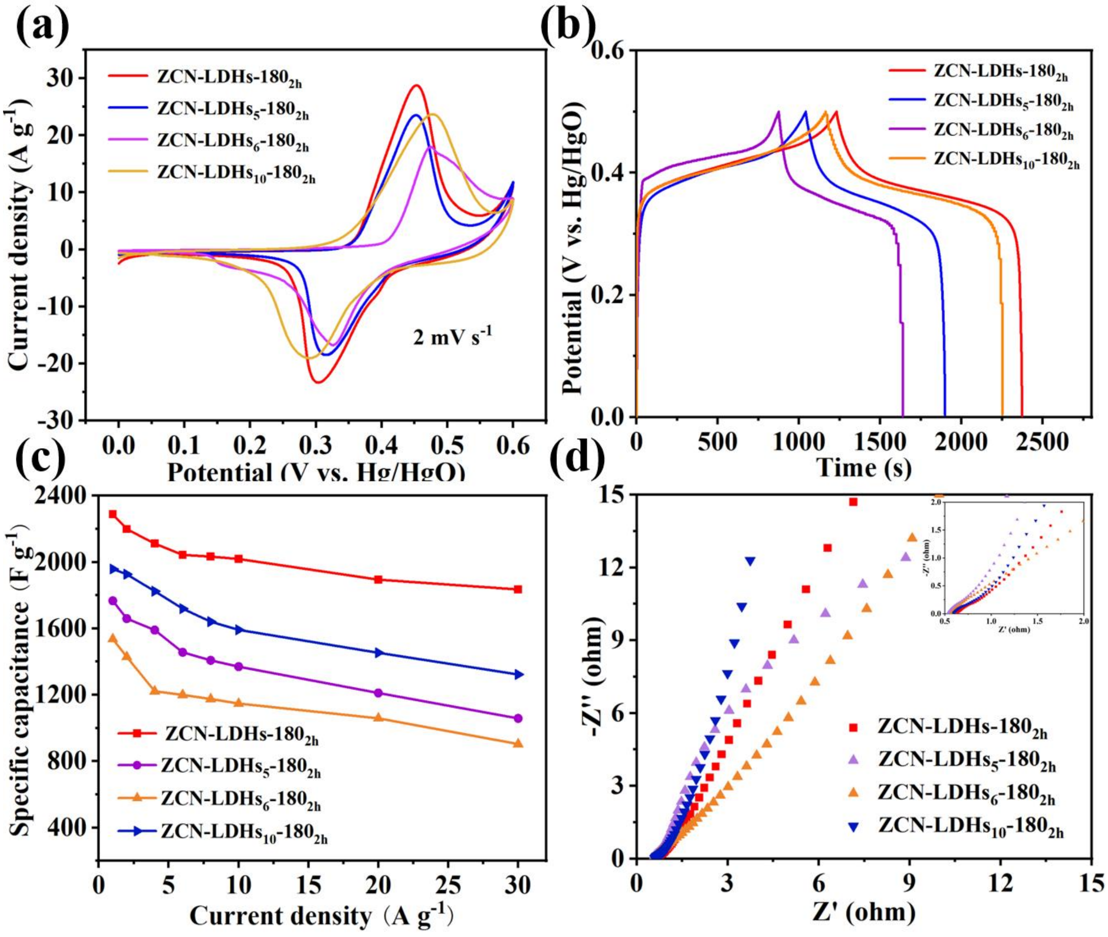

Fig. S12 Samples of ZCN-LDHs-180$_{2h}$, ZCN-LDHs$_5$-180$_{2h}$, ZCN-LDHs$_6$-180$_{2h}$, and ZCN-LDHs$_{10}$-180$_{2h}$: (a) CV curves at a scan rate of 2 mV s^{-1}, (b) GCD curves at a current density of 1 A g^{-1}, (c) Specific capacitance plots at different current densities, and (d) EIS plots.

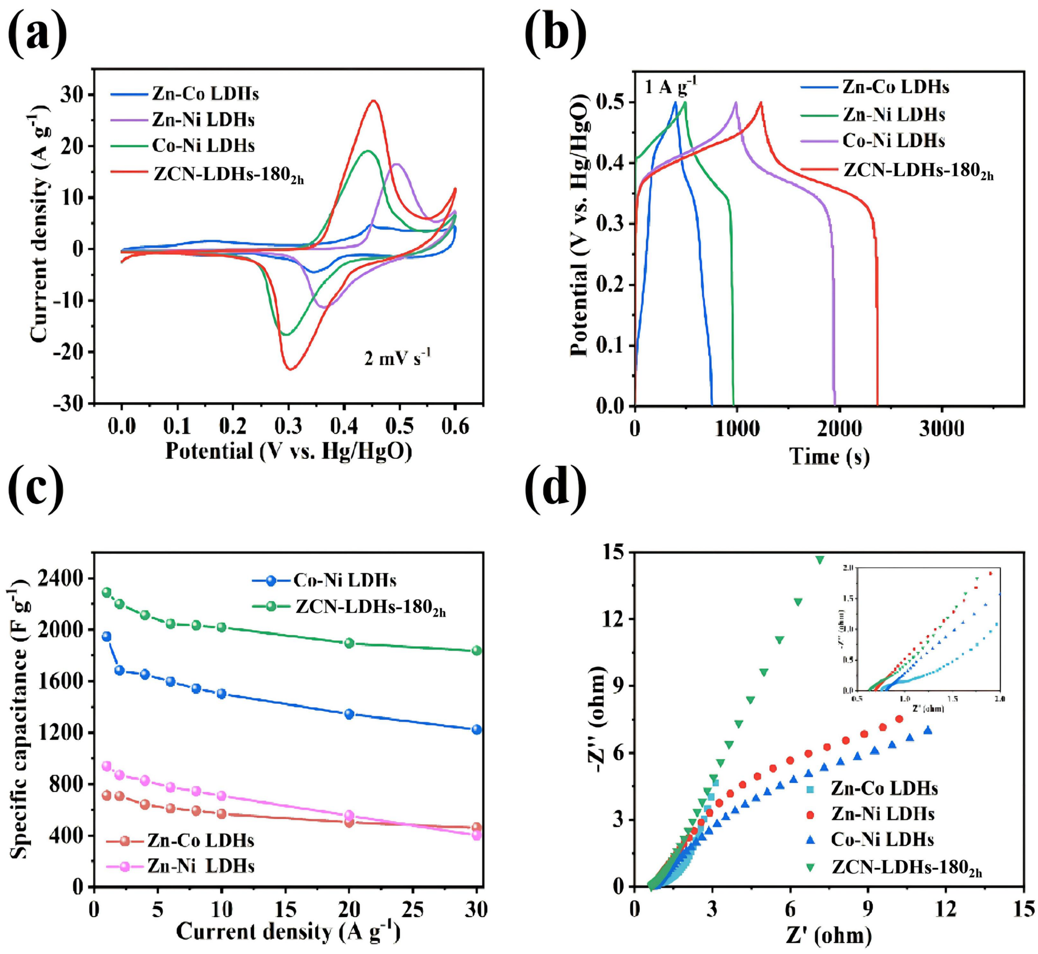

Fig. S13 Samples of ZC-LDHs, ZN-LDHs, CN-LDHs, and ZCN-LDHs-180$_{2h}$: (a) CV curves at a scan rate of 2 mV s^{-1}, (b) GCD curves at a current density of 1 A g^{-1}, (c) Specific capacitance plots at different current densities, and (d) EIS plots.

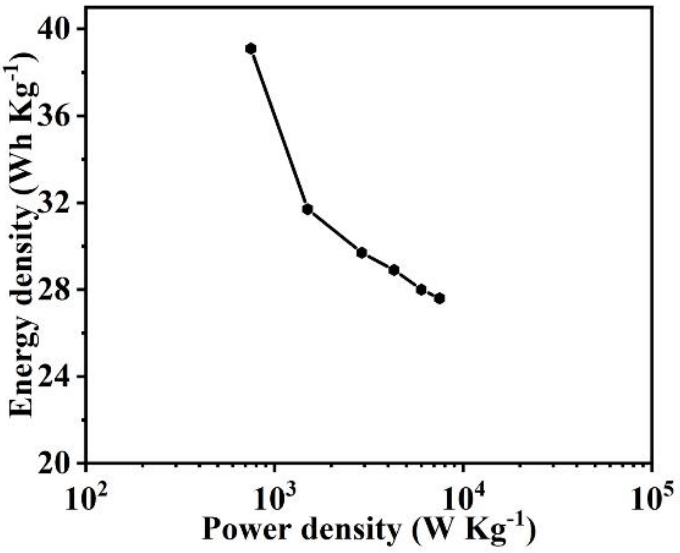

Fig. S14 Ragone plot of ZCN-LDHs-180$_{2h}$//AC.

Table S1 Performance comparison of this work with other similar works.

Materials	Capacity (F g^{-1})	Capacity retention	Ref
Co-Ni LDH	1634.1 (1 A g^{-1})	81.7% (10000 cycles)	1
Co_3O_4@CoNi-LDH	2676.9 (1 A g^{-1})	67.7% (10000 cycles)	2
Mg-Co-Al LDHs/rG-x	1204 (1 A g^{-1})	90.5% (20000 cycles)	3
CoNi LDH@rGO@$CoNi_2S_4$	2101 (1 A g^{-1})	72% (6000 cycles)	4
MnCo-LDH/S-NiCo-LDH@NF	1581.3 (1 A g^{-1})	87% (10000 cycles)	5
NiFeAl LDHs	1652.2 (0.5 A g^{-1})	54.9% (1000 cycles)	6
ZCN-LDHs-180_{2h}	2288 (1 A g^{-1})	84.8% (12000 cycles)	This work

Table S2. Comparison of energy densities between this work and others.

Materials	Energy density (Wh kg^{-1})	Power density (W kg^{-1})	Ref
ZnCoS/ZnCoLDH//AC	36.4	850	7
ZnCo LDH@Ni_3S_2//AC	39	423	8
Co(OH)F/$Ni(OH)_2$//AC	13.8	470	9
NiCo LDH-C_3N_4//AC	33	401	10
Mg-Co-Ni LDHs//AC	44.3	800	3
CoNi LDH NFs//AC	30.1	748	11
ZCN-LDHs-180_{2h}//AC	39.1	750	This work

References

1 Z. Song, Q. Meng, F. Wei, Q. Yin, Y. Sui, J. Qi, *J. Electroanal. Chem.*, 2023, **936**, 117379.

2 J.-J. Zhou, Q. Li, C. Chen, Y.-L. Li, K. Tao, L. Han, *Chem. Eng. J.*, 2018, **350**, 551-558.

3 Y. Yao, Y. Yu, L. Wan, C. Du, Y. Zhang, J. Chen, M. Xie, *J. Colloid Interface Sci.*, 2023, **649**, 519-527.

4 J. Hu, L. Sun, F. Xie, Y. Qu, H. Tan, X. Shi, J. Qian, K. Wang, Y. Zhang, *J. Mater. Chem. A*, 2022, **10**, 21590-21602.

5 Y.-C. Hsiao, C.-H. Liao, C.-S. Hsu, S. Yougbaré, L.-Y. Lin, Y.-F. Wu, *J. Energy Storage*, 2023, **57**, 106171.

6 B. Ramulu, J.A. Shaik, A.R. Mule, J.S. Yu, *Mater. Sci. Eng. R Rep.*, 2024, **160**, 100820.

7 Y. Cao, J. Wang, L. Zhong, J. Zhou, A. Fang, Q. Wang, Y. Zhao, J. Li, J. Gong, Y. Dai, *J. Energy Storage*, 2025, **110**, 115250.

8 F. Vahedizadeh, S. Moraveji, L. Fotouhi, M. Zirak, S. Shahrokhian, *J. Energy Storage*, 2024, **94**, 112460.

9 X. Li, R. Ding, W. Shi, Q. Xu, D. Ying, Y. Huang, E. Liu, *Electrochim. Acta*, 2018, **265**, 455-473.

10 Y. Tan, Y. Ren, Y. Zhang, *J. Energy Storage*, 2024, **81**, 110367.

11 E. Bao, X. Ren, Y. Wang, Z. Zhang, C. Luo, X. Liu, C. Xu, H. Chen, *J. Energy Storage*, 2024, **82**, 110535.
